# Synaptopodin is required for long-term depression at Schaffer collateral-CA1 synapses

**DOI:** 10.1186/s13041-024-01089-3

**Published:** 2024-04-02

**Authors:** Yanis Inglebert, Pei You Wu, Julia Tourbina-Kolomiets, Cong Loc Dang, R. Anne McKinney

**Affiliations:** 1 Department of Pharmacology and Therapeutics, McGill University, Montreal, Canada; 2 Current address Department of Neurosciences, Montreal University, Montreal, Canada

**Keywords:** Synaptopodin, Synaptic plasticity, hippocampus, STDP, LTD, IP3

## Abstract

Synaptopodin (SP), an actin-associated protein found in telencephalic neurons, affects activity-dependant synaptic plasticity and dynamic changes of dendritic spines. While being required for long-term depression (LTD) mediated by metabotropic glutamate receptor (mGluR-LTD), little is known about its role in other forms of LTD induced by low frequency stimulation (LFS-LTD) or spike-timing dependent plasticity (STDP). Using electrophysiology in ex vivo hippocampal slices from SP-deficient mice (SPKO), we show that absence of SP is associated with a deficit of LTD at Sc-CA1 synapses induced by LFS-LTD and STDP. As LTD is known to require AMPA- receptors internalization and IP3-receptors calcium signaling, we tested by western blotting and immunochemistry if there were changes in their expression which we found to be reduced. While we were not able to induce LTD, long-term potentiation (LTP), albeit diminished in SPKO, can be recovered by using a stronger stimulation protocol. In SPKO we found no differences in NMDAR, which are the primary site of calcium signalling to induce LTP. Our study shows, for the first time, the key role of the requirement of SP to allow induction of activity-dependant LTD at Sc-CA1 synapses.

## Introduction

Synaptopodin (SP) is a postsynaptic actin-associated protein found at the excitatory synapses of a subset of mature dendritic spines [1]. It is primarily expressed in the principal neurons of the hippocampus and cortex, which are regions of the brain that are highly plastic [2]. Previous studies have shown that SP is involved in synaptic plasticity and dendritic spine remodeling at these brain regions [3, 4]. Lack of SP results in a reduced NMDA-dependent long-term potentiation (LTP) induced by high frequency stimulation, deficits in AMPA-receptors trafficking and spatial learning [5–7]. It is also necessary for the formation and stabilization of the spine apparatus (SA), an organelle structure which is a source and a regulator of intracellular calcium stores [8, 9]. Recently, our group demonstrated that SP is required for long-term depression (LTD) mediated by group I metabotropic glutamate receptor (mGluR-LTD) [10, 11]. However, it is unclear whether SP is also required for other forms of activity-dependant LTD such as induction by low-frequency stimulation (LFS) and Spike-Timing Dependent Plasticity (STDP). The first is part of a theoretical framework (Bienenstock-Cooper-Munro or BCM rule) describing how synaptic plasticity can be modulated by the level of neuronal activity and the history of synaptic inputs [12]. In this paradigm, low-frequency stimulation (2–5 Hz) induces LTD (LFS-LTD) while high-frequency stimulation (10–100 Hz) induces LTP (HFS-LTP) but the threshold between LTD/LTP can be dynamically adjusted. Both require an increase in intracellular calcium (Ca^2+^) from NMDA receptors (NMDAR), at least at CA3-CA1 synapses [13]. While some studies suggested that only spines containing SP are more likely to develop LFS-LTD [14], others reported a normal LFS-LTD in SP-deficient mice [15].

The second model STDP, thought to be a more physiological way of inducing synaptic plasticity, rather than being dependent on the stimulation frequency, synaptic modifications are based on the precise timing between presynaptic (in the form of excitatory postsynaptic potential, (EPSP)) and postsynaptic (in the form of an action potential ( AP)) activity. These paradigms are thought to better mimic what happens in the brain [16]. Classically, timing-dependent long-term potentiation (t-LTP) is induced when an EPSP is followed by an AP in the postsynaptic neuron [17, 18]. In contrast, timing-dependent long-term depression (t-LTD) is induced when an EPSP is preceded by an AP [19–21]. At CA3-CA1 synapses, both induction protocols require intracellular calcium elevation, but while t-LTP requires strong Ca^2+^ entry from NMDA- and AMPA-receptors (AMPAR) activation, t-LTD relies on moderate Ca^2+^ release from internal calcium stores through IP3 receptors (IP3R) triggered by mGluR5 activation [22, 23]. This is a general learning rule underlying memory found in vitro and in vivo in a wide range of species [24] but to date no study has explored the role of SP in STDP.

In this study, to better understand the role of SP at Schaffer collateral-CA1 synapses (Sc-CA1), we induced LFS-LTD/HFS-LTP or t-LTD/t-LTP in wild-type (WT) and SP-deficient mice (SPKO). Our results show that in the absence of SP, it is impossible to induce LFS-LTD or t-LTD. Nevertheless, although affected, t-LTP can be recovered by boosting Ca^2+^ entry through specific activity patterns. These results provide novel insight into the role of SP in LTD and describe SP as a key molecular component to enable synaptic plasticity.

## Results

### Synaptopodin is required for LTD induced by low frequency stimulation

**Fig. 1:**
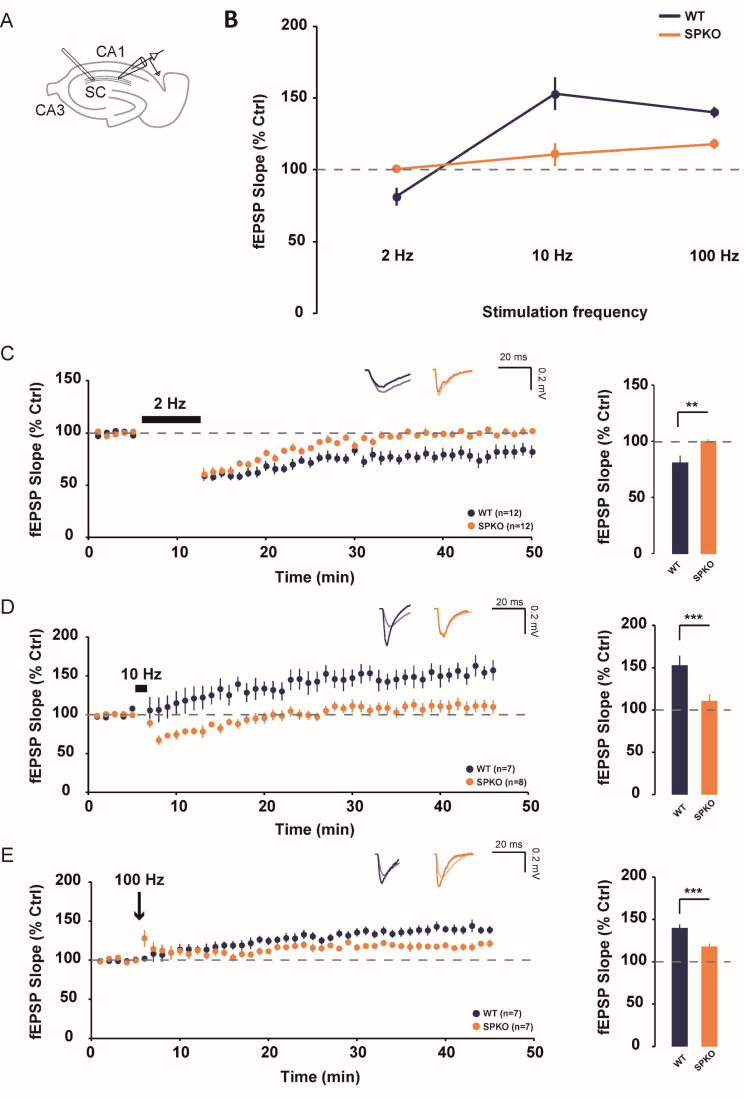
SPKO mice show a deficit of LFS-LTD and HFS-LTP. (**A**) Diagram showing the positions of stimulating and recording electrodes at the Sc-CA1 synapses. (**B**) BCM curves in WT and SPKO for 2, 10 and 100 Hz stimulation frequency. (**C**) *(Left)* Time course of normalized fEPSP in WT and SPKO following 2 Hz (900 pulses) stimulation. *(Right)* Quantification of average synaptic modification in the last 10 min of the recording. (**D**) *(Left)* Time course of normalized fEPSP in WT and SPKO following 10 Hz (900 pulses) stimulation. *(Right)* Quantification of average synaptic modification in the last 10 min of the recording. (**E**) *(Left)* Time course of normalized fEPSP in WT and SPKO following 100 Hz (900 pulses) stimulation. *(Right)* Quantification of average synaptic modification in the last 10 min of the recording

Firstly, we wanted to determine the consequences of the absence of SP on synaptic plasticity induced by either low- or high-frequency stimulation to gain an overview of the BCM curve. The Schaffer collaterals were stimulated with 900 pulses at 2, 10 or 100 Hz (Fig. 1A). LFS-LTD and HFS-LTP are known to be induced respectively by 2 and 100 Hz while 10 Hz is generally the threshold between LTD/LTP. In SPKO, no significant change in synaptic modification (field EPSP slope measured on the last 10 min of the recording) was observed after 2 Hz (100.3 ± 1.7 vs. 81.4 ± 6.1%, *p* < 0.003, Mann-Whitney) and 10 Hz (110.68 ± 7.8 vs. 152.96 ± 11.1%, *p* < 0.03, Mann-Whitney) stimulation compared to WT (Fig. 1C, D). In contrast, WT exhibited a robust LFS-LTD and HFS-LTP respectively for 2 Hz and 10 Hz stimulation. At 100 Hz, SPKO revealed a modest HFS-LTP in comparison to the magnitude found in WT (Figs. 1E and 117.9 ± 3.4 vs. 140.1 ± 3.7%, *p* < 0.001, Mann-Whitney). Overall, in SPKO, our data show that LFS-LTD is absent and HFS-LTP amplitude is decreased (Fig. 1B) and the threshold for synaptic changes is higher in the absence of SP.

### Spike-timing dependent plasticity is altered in absence of synaptopodin

**Fig. 2:**
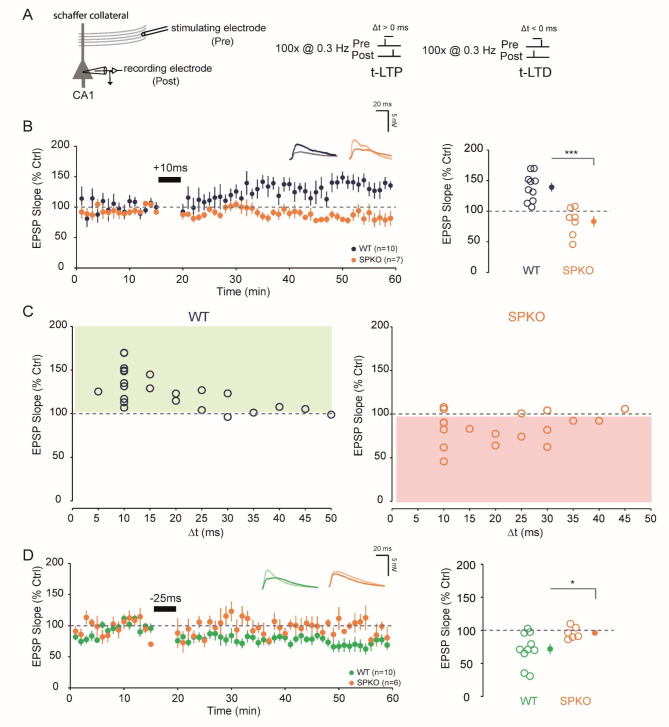
Spike-Timing Dependent Plasticity is altered in SPKO. (**A**) *(Left)* Diagram showing the positions of stimulating electrodes and recording electrodes at the Sc-CA1 synapses. *(Right)* Illustration of the pre-before-post (100p@0.3 Hz) and post-before-pre (100p@0.3 Hz) protocol used to induce synaptic modification. (**B**). *(Left)* Time course of normalized EPSP in WT and SPKO following pre-before-post protocol at + 10ms. *(Right)*. Quantification of average synaptic modification (*closed circles*) in the last 10 min of the recording with individuals’ values for each cell (*open circles*). (**C**). STDP curves in WT and SPKO for pre-before-post protocol at different timings. (**D**). *(Left)* Time course of normalized EPSP in WT and SPKO following post-before-pre protocol at -25ms. *(Right)*. Quantification of average synaptic modification (*closed circles*) in the last 10 min of the recording with individuals’ values for each cell (*open circles*)

We next examined the consequences of the lack of SP on synaptic plasticity induced at the Sc-CA1 pyramidal cell synapses by STDP (Fig. 2A, Left). Classically, t-LTP was induced by repeatedly pairing (100 times, 0.3 Hz) an EPSP followed briefly (between + 5 and + 50ms, positive pairing) by a postsynaptic action potential (AP) (Fig. 2A, Right). No t-LTP is observed in SPKO at + 10ms, a timing that generally shows the highest t-LTP amplitude in WT. (Figures 2B and 83.3 ± 8,6 vs. 139.2 ± 7,1%, *p* < 0.001, Mann-Whitney). Construction of STDP curves for several timings exhibited a prolonged t-LTD window ranging from + 10ms to + 30ms in SPKO (Fig. 2C, Right) while the conventional LTP window is observed in WT (Fig. 2C, Left). For negative pairing (a postsynaptic AP followed by an EPSP) at -25ms, a timing that generally shows the highest t-LTD amplitude in WT, no plasticity is observed in SPKO (Figs. 2D and 96.2 ± 3.7 vs. 71.9 ± 7.7%, *p* < 0,05, Mann-Whitney). These data indicate that the STDP rule is severely impaired in the absence of SP and t-LTD is totally absent for negative pairings.

### t-LTP but not t-LTD can be rescued in absence of synaptopodin

**Fig. 3:**
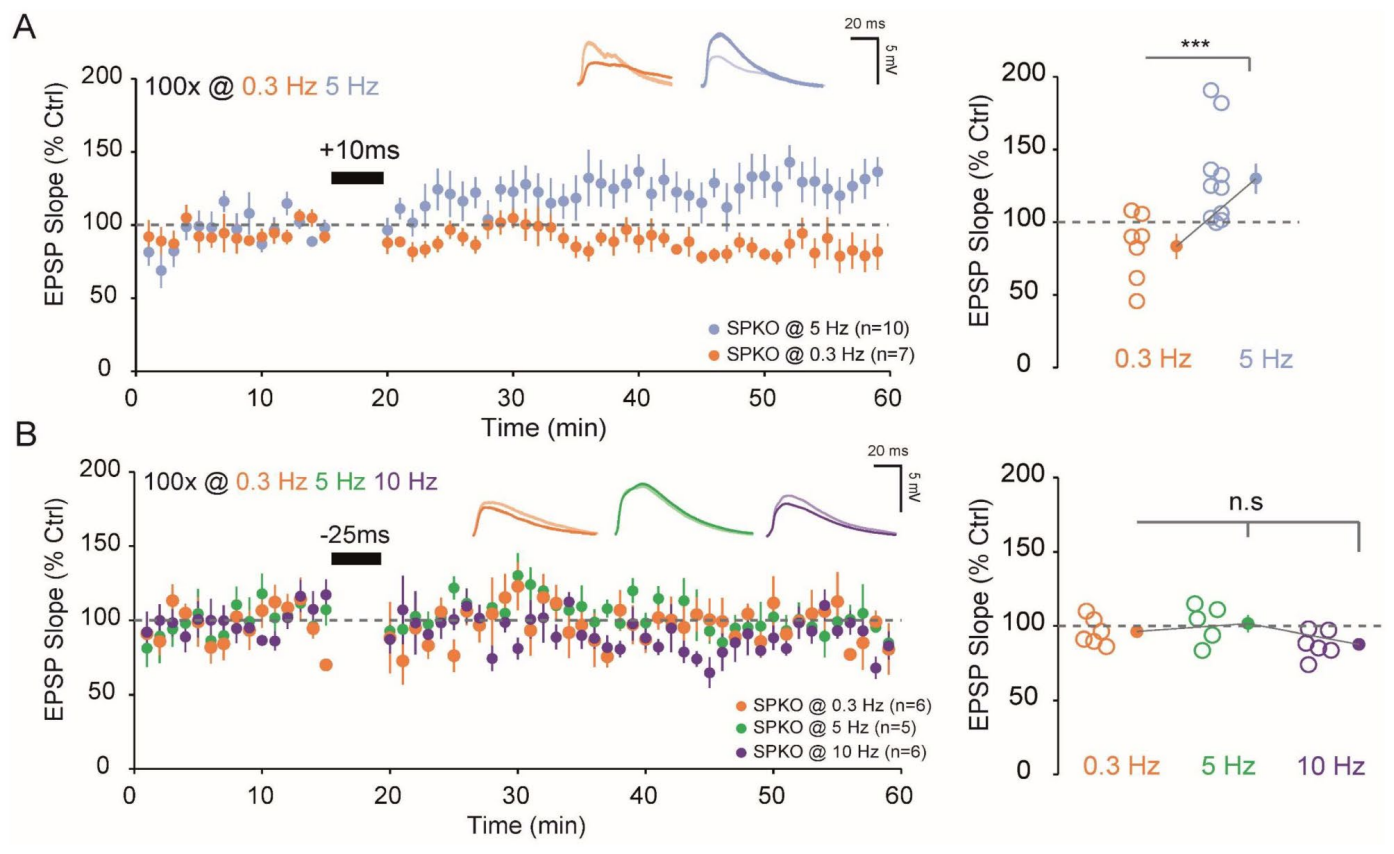
t-LTP but not t-LTD can be restored in SPKO. (**A**). *(Left)* Time course of normalized EPSP in WT and SPKO following pre-before-post (+ 10ms) protocol at 0.3–5 Hz. *(Right)*. Quantification of average synaptic modification (*closed circles*) in the last 10 min of the recording with individuals’ values for each cell (*open circles*). (**B**). *(Left)* Time course of normalized EPSP in WT and SPKO following post-before-pre (-25ms) protocol at 0.3, 5–10 Hz. *(Right)*. Quantification of average synaptic modification (*closed circles*) in the last 10 min of the recording with individuals’ values for each cell (*open circles*)

STDP induction rules are flexible and can be adapted by modulating intracellular Ca^2+^ influx [25–27]. For example, an easy way to boost intracellular Ca^2+^ influx is to increase the stimulation frequency which would lead to cross the synaptic threshold for t-LTD and t-LTP. Taking this into account we attempted to recover t-LTP and t-LTD deficits in SPKO by increasing the pairing frequency. At + 10ms, increasing the pairing frequency from 0.3 to 5 Hz has been sufficient to significantly recover the t-LTP window (Fig. 3A, 129.9 ± 10.3%, *p* < 0,05, Mann-Whitney). On the other hand, increasing the pairing frequency did not significantly recover t-LTD window at -25ms neither at 5 Hz (101.6 ± 5.7%) nor at 10 Hz (87.7 ± 3.7%) compared to 0.3 Hz (Fig. 3B). Similarly to the results obtained in Fig. 1, t-LTP, while affected, can still be induced but t-LTD remained totally absent. We concluded that t-LTP can be rescued in SPKO but not t-LTD.

### AMPA and IP3-receptors expression is altered in SPKO

**Fig. 4:**
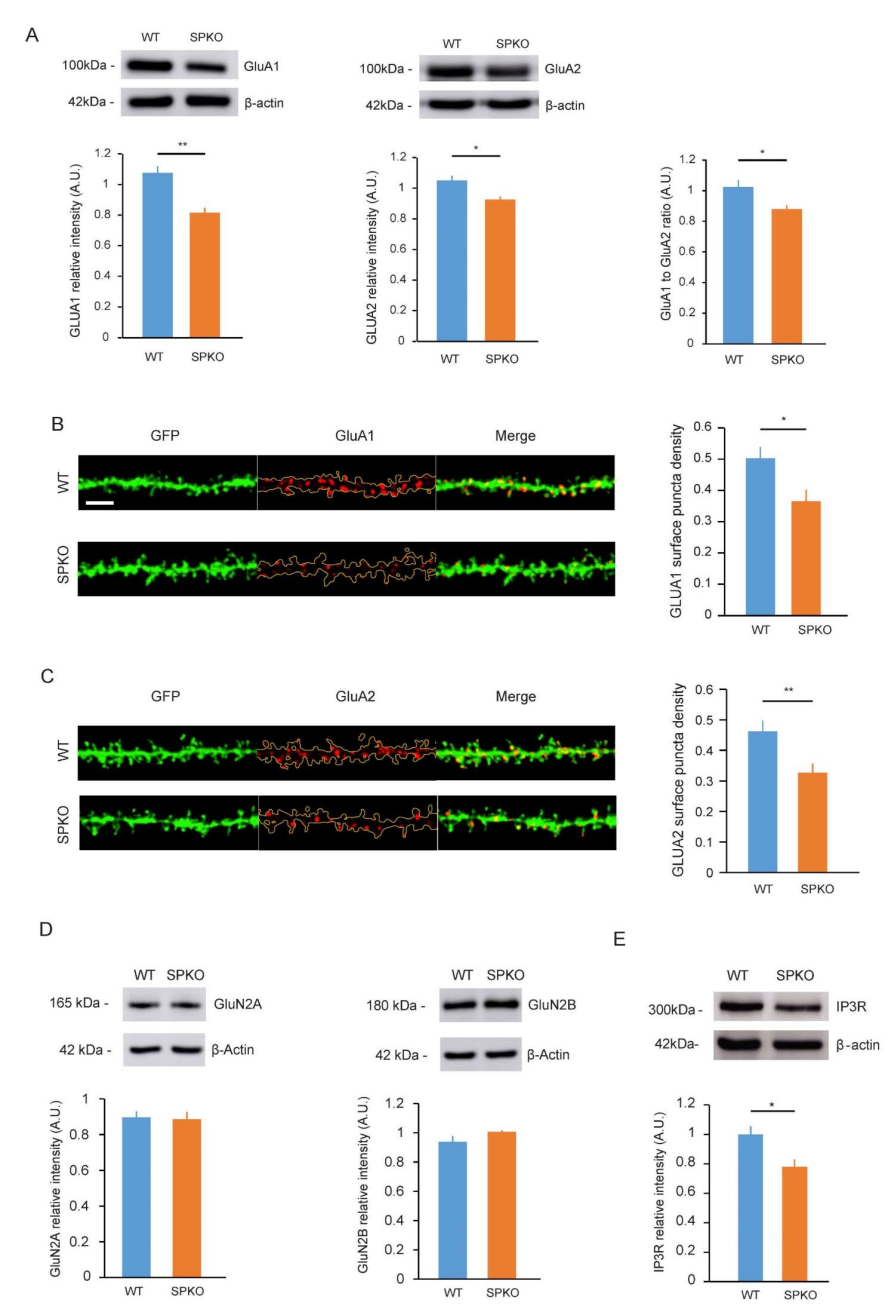
GluA1, GluA2 and IP3R are decreased in SPKO. (**A**). Representative immunoblot and quantification of total (*left*) GluA1 and (*right*) GluA2 from hippocampi of WT (*n* = 5) and SPKO (*n* = 5) mice. (**B**, **C**). Representative images and quantification of CA1 pyramidal neuron tertiary dendrites immunostained with surface GluA1 antibody (WT *n* = 12, KO *n* = 14 slices) and surface GluA2 antibody (WT *n* = 13, KO *n* = 11 slices) in acute hippocampal slices. Scale bar = 3 μm. (**D**). Representative immunoblot and quantification of total (left) GluN2A and (right) GluN2B from hippocampi of WT (*n* = 5) and SPKO (*n* = 5) mice. (**E**). Representative immunoblot and quantification of total IP3R from hippocampi of WT (*n* = 8) and SPKO (*n* = 8) mice

To better understand what could lead to these deficits of synaptic plasticity in SPKO, we examined the expression level of the key receptors involved in these two forms of synaptic plasticity by Western blot from hippocampi lysates or immunohistochemistry of WT and SPKO. We initially investigated if there were changes in the total protein of GluA1/GluA2 subunits of AMPARs [28, 29] in SPKO vs. WT hippocamapi. We observed a significant decrease in total protein of AMPA-receptor subunits GluA1 (1.07 ± 0.04 vs. 0.81 ± 0.03 A.U, *p* < 0.01, unpaired t-test) and GluA2 (1.05 ± 0.03 vs. 0.92 ± 0.02 A.U, *p* = 0.01, unpaired t-test) in SPKO compared to WT (Fig. 4A). Using immunohistochemistry we tested for changes in surface GluA1 and GluA2 at the surface of tertiary dendrites of membrane tagged-GFP CA1 pyramidal neurons in acute hippocampal slices in WT and SPKO. We found a significant reduction of the surface expression of GluA1 (Fig. 4B, 0.50 ± 0.04 vs. 0.37 ± 0.04 A.U, *p* < 0.05, unpaired t-test) and GluA2 (Fig. 4C, 0.46 ± 0.04 vs. 0.33 ± 0.03 A.U, *p* < 0.01, unpaired t-test). Using Western blot analysis, we also tested for changes in total protein GluN2A/GluN2B subunit of NMDARs because they are the major subunits found in the hippocampus. In contrast, no difference in expression was found for NMDA receptor subunits GluN2A (Fig. 4D Left, 0.90 ± 0.03 vs. 0.89 ± 0.04 A.U, *p* > 0.05, unpaired t-test) and GluN2B (Fig. 4D Right, 0.94 ± 0.04 vs. 1.01 ± 0.01 A.U, *p* > 0.05, unpaired t-test). We found a significantly decreased in total protein in IP3-receptors expression in SPKO (Fig. 4E, 1.00 ± 0.06 vs. 0.78 ± 0.05 A.U, *p* < 0.05, unpaired t-test). Overall, IP3- and AMPA- receptors expression was found to be significantly reduced unlike NMDA receptors in SPKO.

## Discussion

### LFS-LTD is absent in SPKO

Our results revealed a lack of LFS-LTD in SPKO compared to WT (Fig. 1). Our findings contradict previously published findings [15] that LFS-LTD was not modulated by loss of SP. The experimental conditions are similar regarding the stimulation protocol (2 Hz 1200 stimuli vs. 2 Hz 900 stimuli) and the age of mice (P14-P25). An attractive explanation for this difference could be that the LFS-LTD threshold is higher in SPKO. In fact, previous studies have shown a relationship between the number of stimuli and LTD amplitude [30]. In line with this concept, it has been shown that in the hippocampus, all synapses have a different threshold for LTP and LTD [31, 32]. More importantly, 70% of CA1 synapses have been shown to be already in a “depressed” state making it often more difficult to induce LTD [33]. Another possible explanation is that the extracellular calcium concentrations used the experiments are different. While we used a more physiological concentration of calcium (2mM), Zhang et al., employed a concentration of 2.5mM. Higher calcium concentrations could lead to overestimation of plasticity [26, 34]. Finally, SP has been shown to be involved in AMPA-receptors trafficking to the membrane following LTP [7]. It is possible that the absence of SP could prevent proper internalization of AMPA- receptors which normally occurs during LTD. Additional future experiments will be required to test this hypothesis.

### BCM curve is shifted in SPKO

In SPKO slices, our data showed that at 10 Hz, HFS-LTP was absent. 10 Hz is a particularly interesting frequency, as it is considered to be the threshold (θm) between LTD and LTP [12]. This threshold is dynamic, slidable and can serve as a homeostatic mechanism to maintain synapses within a modifiable zone in response to perturbations in neuronal activity. Interestingly, lack of SP has been linked to a loss of GABAergic inhibition [35] and increased excitability [36]. To compensate for this increase in CA1 cell excitability, the sliding threshold could horizontally shift to the right as demonstrated in other brain regions [37]. It is also important to considerer the role of SP in metaplasticity which refers to a neuron’s capacity to modify its plasticity threshold [38]. SP has been linked to metaplasticity regulation in the hippocampus in the dentate gyrus. Loss of SP prevented Tumor Necrosis Factor (TNF) and all-trans retinoic acid (atRA) to promote metaplasticity in CA1 and the dentate gyrus, respectively [39, 40]. SP has also recently been identified as a molecular tag to promote input-specific homeostatic plasticity in the hippocampus [41]. Further studies are required to better understand the role of SP in homeostatic plasticity at Sc-CA1 synapses.

### t-LTP can be rescued but not t-LTD

At Sc-CA1 synapses, both t-LTP and t-LTD require post-synaptic calcium (Ca^2+^) elevation [42, 43] but from different sources. The magnitude of the Ca^2+^ signal determines the direction/polarity of plasticity [44]. Whereas t-LTP relies almost solely on Ca^2+^ entry via post-synaptic NMDA receptors (NMDAR), t-LTD required activation of mGluR5 and subsequent IP3R-mediated Ca^2+^ release from internal stores [22] and both are affected by the absence of SP. As we previously reported, mGluR5 function and expression is impaired [11]. Moreover, SP is known to regulate release of Ca^2+^ from internal stores [45] and our results indicate that IP3R expression is reduced. In other words, if t-LTD is absent and impossible to recover, it’s certainly because the needed molecular machinery is not present in the absence of SP. On the other hand, t-LTP can simply be recovered in SPKO by increasing the pairing frequency (from 0.3 to 5 Hz) and therefore the post-synaptic calcium entry. This finding is supported by the idea that the STDP rule is flexible.

Adjusting parameters such as the number of repetitions, the number of post-synaptic action potentials or the pairing frequency can facilitate the induction of LTP by boosting post-synaptic calcium influx [27, 31, 46, 47].In addition to NMDAR, AMPA receptors mediated EPSPs provide local depolarization to boost NMDAR current [48, 49]. A reduction of AMPAR expression could reduce NMDAR current for a given depolarization, explaining the absence of t-LTP observed at 0.3 Hz. A stronger protocol (5 Hz) overcomes the impact of AMPAR reduction and enables the potentiation threshold to be crossed as NMDAR expression is not affected in SPKO, and the calcium entry provided by NMDAR only is now sufficient. Alternatively, the post-synaptic cell may be depolarized to boost calcium entry through NMDA- receptors and recover t-LTP [50]. Additional calcium imaging experiments will be required to better understand the calcium dynamics at the level of the single spine-expressing SP.

### AMPA receptors expression

Surprisingly, we found that AMPAR expression (GluA1 and GluA2 subunits) and surface expression are reduced in SPKO. That would seem perfectly logical because SP-positive spines have been shown to express more AMPAR receptors compared to SP-negative spines [7]. On the other hand, previous studies performed in the dentate gyrus [4, 51] and dissociated cultured of hippocampus have shown no difference in basal synaptic transmission [3]. The role of SP in AMPAR synaptic stabilization is likely activity dependent [7, 41]. Following LTP or homeostatic plasticity, SP is required for the recruitment and stabilization of AMPARs. Although the amount of AMPAR at the synapse is normal between SPKO and WT, the pool of “ready to be addressed” AMPAR (such as perisynaptic AMPAR) are probably reduced. Unfortunately, western blot and immunohistochemistry have their limitations. It is important to note that our western blot is performed on the whole hippocampi, and we cannot exclude differences in AMPAR expression in another area (CA2, CA3 for example). Additional studies using super-resolution microscopy (STED) will help to better understand the AMPAR dynamics in absence of SP.

### STDP to explore homo- and hetero- plasticity

Because STDP is known to exist at the level of the single spine [52], it is the perfect framework to study homo- and heterosynaptic plasticity. It has been shown that following glutamate uncaging on SP-positive spine, neighbouring synapses exhibit a spine head shrinkage [53]. This suggests a role for SP in both homo- and heterosynaptic plasticity. Further studies will be required to better characterize this type of plasticity in the context of STDP.

### Synaptopodin during development

It appears that SP is only required for normal synaptic plasticity in the developing hippocampus. Indeed, lack of SP has been linked to deficit in LTP in younger (2–8 weeks) but not in adult animals (2 to 6-month-old). It is most likely explained by an increase in intrinsic excitability to compensate and restore LTP [15]. Furthers studies need to address the intrinsic properties of CA1 pyramidal neurons in SPKO, especially because SP is also express in the axon initial segment (AIS) and could profoundly modify excitability and AIS development [54]. Additionally, in SPKO mice, spine density is normal but dendritic spines turnover is increased [55], and it is not clear how this higher spine dynamics could affect hippocampal development and network maturation.

Our results demonstrate for the first time the central role of SP in LTD, particularly induced by STDP in the developing hippocampus. These findings encourage further exploration and dissection of the role of SP in plasticity at the scale of the single spine. It could be the key to understanding why dendritic spines are not all equal when it comes to plasticity.

## Materials and methods

### Animals

C57Bl6 (WT) or SPKO mice were used as previously described [3, 27]. Mice were fed *ad libitum* and housed with a 12 h light/dark cycle. WT and SPKO male and female mice were used for experiments.

### Electrophysiology

Hippocampal ex vivo slices were obtained from P14-P25 old WT or SPKO mice. Mice were deeply anesthetized with isoflurane and killed by decapitation. Slices (400 μm–350 μm) were cut on a vibratome (Leica Microsystems, VT1200S) in a sucrose-based solution containing the following (in mM): 280 sucrose, 26 NaHCO3, 10 glucose, 1.3 KCl, 1 CaCl2, and 10 MgCl2 and were transferred at 32 °C in regular ACSF containing the following (in mM): 124 NaCl, 5 KCl, 1.25 NaH2PO4, 2 MgSO4, 26 NaHCO3, 2 CaCl2, and 10 glucose saturated with 95% O2/5% CO2 (pH 7.3, 300 mOsm) for 15 min before resting at room temperature (RT) for 1 h in oxygenated (95% O2/5% CO2) ACSF. Recordings were obtained using a Axopatch 200B (Molecular Devices) amplifier and pClamp10.4 software. Data were sampled at 10 kHz, filtered at 3 kHz, and digitized by a Digidata 1440 A (Molecular Devices). Field excitatory postsynaptic potentials (fEPSPs) or excitatory postsynaptic potentials (EPSPS) were elicited in the *stratum radiatum* of the CA1 region by using glass microelectrodes filled with 3 M NaCl. fEPSPs or EPSPs were elicited at 0.1 Hz by a digital stimulator that fed by a stimulation isolator unit. All data analyses were performed with custom-written software in Igor Pro 8 (Wavemetrics). fEPSP or EPSP slope was measured as an index of synaptic strength. For whole-cell recordings, access resistance was monitored throughout the recording and only experiments with stable resistance were kept (changes < 20%). Recordings were made from CA1 pyramidal neurons, electrodes were filled with a solution containing the following (in mM): 120 K-gluconate, 20 KCl, 10 HEPES, 2 MgCl26H2O, and 2 Na2ATP.

### Immunohistochemistry

Hippocampal slices of 100 μm were obtained from P30 to P40 old L15S or L15 mice. The slices were incubated in ACSF at RT for 1 h for recovery and fixed in 0.1 M Phosphate buffer (PB) containing 4% PFA, pH 7.4, overnight at 4 °C. After fixation, slices were washed in 0.1 M PB and blocked with 1.5% heat-inactivated horse serum overnight at 4 °C. Slices were incubated with primary anti-GluA1 extracellular antibody (1:200, Alomone Lab, CAT# AGC-004) and primary anti-GluA2 extracellular antibody (1:200, Alomone Lab, CAT# AGC-005) in blocking solution for 5 days at 4 °C, washed with PB, and incubated with anti-rabbit secondary antibody conjugated to DyLight 649 (1:500; Jackson ImmunoResearch Laboratories) overnight. They are then washed and mounted with DAKO Fluorescent Mounting medium (Dako Canada) onto microscope slides before imaging and subsequent blinded analysis. Spine images were taken in z stacks using a Leica Microsystems SP8 confocal microscope with oil-immersion 63x objective at 6x zoom. The images were deconvolved and analyzed using software Huygens (Scientific Volume Imaging) and Imaris Software (Oxford Instruments) respectively. The experimenter was blinded to the treatment.

### Immunoblotting

Hippocampi of WT and SPKO mice at P20 were obtained and rapidly homogenized in ice-cold RIPA (150 mM NaCl, 1% Nonidet P-40, 0.5% sodium deoxycholate, 0.1% sodium dodecylsulfate, 50 mM Tris-HCl (pH 8.0) with protease and phosphatase inhibitors (1X Roche Complete Mini, 5 mM NaF, 1 mM sodium orthovandate, 1 mM PMSF). Homogenates were sonicated and centrifuged for 5 min at 13,200 rpm. The supernatant was extracted and mixed with 2x Laemmli sample buffer to produce loading samples. Samples were boiled, resolved on SDS-PAGE, transferred to nitrocellulose, and stained with primary antibodies overnight. The following primary antibodies were used: GluA1 (1:1000, Abcam, ab31232), GluA2 (1:1000, Abcam, ab20673), GluN2A (1:1800, Abcam, ab124913,), GluN2B (1:1800, Abcam, ab65783), IP3R (1:1000, Cell Signaling, D53A5). Horseradish peroxidase (HRP)-conjugated secondary antibodies goat anti-rabbit (abcam; 1:5000) or goat anti-mouse (Bio-Rad Laboratories; 1:10,000) were applied for 1 h. The blots were imaged with Amersham Imager 600 and the band intensities were analyzed using the open-source program Fiji.

## Data Availability

All data generated or analysed during this study are included in this published article.

## Preprint

This manuscript was posted on a preprint: 10.1101/2023.08.21.554176
